# Recorded Contingent Caregiver Voice Independently Improves Neural Speech Processing in Hospitalized Preterm Infants: A Multisite Randomized Controlled Trial

**DOI:** 10.3390/jcm15176880

**Published:** 2026-09-05

**Authors:** Caitlin P. Kjeldsen, Megan Moran, Arnaud Jeanvoine, Joshua Lukemire, Gordon Ramsay, A. Joselyn Barahona, Nathalie L. Maitre

**Affiliations:** 1 School of Medicine, Emory University, 2220 North Druid Hills Road NE, Atlanta, GA 30329, USA; caitlin.kjeldsen@emory.edu (C.P.K.);; 2 Children’s Healthcare of Atlanta, 2015 Uppergate Drive, Atlanta, GA 30322, USA; 3 Harmonips, LLC, 645 South Cassingham Road, Columbus, OH 43209, USA; 4 Department of Biostatistics and Bioinformatics, Rollins School of Public Health, Emory University, 1518 Clifton Road NE, Atlanta, GA 30322, USA; 5 Marcus Autism Center, School of Medicine, Emory University, 1920 Briarcliff Road NE, Atlanta, GA 30329, USA

**Keywords:** infant-directed voice, contingent learning, preterm infant, neurodevelopment, NICU, auditory ERP, speech sound discrimination

## Abstract

**Background/Objectives**: The atypical NICU auditory environment can disrupt neural plasticity critical for language development in preterm infants. Recorded caregiver’s voice offers input when bedside presence is infeasible, and evidence suggests contingency, delivering the recording in response to infant behavior, is key to its benefit. This study tested whether caregiver’s recorded voice contingent on non-nutritive sucking (NNS) enhances neural speech processing beyond passive exposure. **Methods**: This randomized controlled trial enrolled preterm infants born before 35 weeks gestational age (GA), and between 32 0/7–35 6/7 weeks corrected GA at start. Infants were randomized to an intervention group receiving caregiver’s voice contingent on NNS, or a control group receiving passive voice exposure. Primary outcomes were post-intervention auditory ERP responses at left (T5) and right (T6) temporal locations, adjusted for pre-intervention EEG. Sensitivity analyses controlled for GA and maternal education; secondary analyses examined bed type, room type, and sound levels. **Results**: Of 214 infants enrolled, 97 intervention and 94 control infants completed the protocol (median GA 31.9 weeks, median corrected GA at start 33.9 weeks). The intervention group showed significantly greater speech-sound processing at T5 (β = 0.023, 95% CI [0.002, 0.044], *p* = 0.036, d = 0.31), with a borderline effect at T6 (β = 0.022, 95% CI [0.000, 0.045], *p* = 0.054, d = 0.28), both strengthened in sensitivity analyses. No significant interactions emerged with bed type, room type, or sound level. **Conclusions**: Contingent, infant-activated caregiver-voice exposure improves auditory ERP responses in preterm infants, independent of environmental factors, suggesting active engagement, not voice alone, as the driver of change. Further studies are warranted.

## 1. Introduction

Preterm infants often experience atypical brain maturation, even in the absence of overt neural insults [1]. This heightened risk for disrupted neurodevelopment can be partially attributed to the atypical sensory environment of the neonatal intensive care unit (NICU) combined with medical complexities inherent to preterm birth [2,3,4,5]. Experience-dependent plasticity in the neonatal period relies on sensory experiences, including auditory input, to construct new neural connections [6]. Without these necessary stimuli, the basic early building blocks of more complex neural processes (e.g., communication, cognition) may be missing or poorly constructed [7].

Because early auditory exposure is linked to communication and language development, the auditory environment of the NICU carries particular weight for these emerging systems [7]. Not all sound is equally meaningful to the developing infant brain and therefore, auditory experience has the potential to both help or harm the developing brain [6]. In utero, fetuses frequently hear their mother’s voice, learning to recognize it above all others at birth, creating the foundation for subsequent language development [8]. For preterm infants, the combined disruption to gestational development and early separation between the infant and their mother reduces access to intrauterine voice exposure [9,10]. Consequently, preterm infants miss a critical developmental opportunity in which maternal voice exposure ordinarily scaffolds language acquisition, supports cognitive development, and facilitates the mutual recognition and attunement essential to bonding [11]. Previous work has shown improved language outcomes following intentional exposure to caregivers’ voices during NICU hospitalization [11,12]; however, barriers to caregivers’ presence at bedside, such as logistical challenges, and emotional and financial stressors associated with early hospitalization, often limit opportunities for infants to hear their caregiver’s voice [13]. Recordings of caregivers’ voices offer a feasible and affordable alternative when caregivers are unable to be consistently present [14,15]. However, the optimal delivery method, dose, duration, and volume for these recordings remain unknown, and it is unclear what parameters are both safe and effective for the developing auditory system.

Growing evidence points to contingency as a key variable in optimizing delivery of recorded caregiver’s voice in the NICU [14,16,17], that is, whether the recording is delivered passively or in response to the infant’s own active behaviors. This form of infant-controlled, contingent auditory reinforcement draws on a robust evidence base demonstrating that infants, as early as 32 weeks gestational age, can reliably engage with and learn from salient auditory stimuli [14,16,17,18]. Recent findings from the MIND randomized controlled trial showed significant improvement in speech-sound differentiation and long-term language outcomes following the multisensory MIND intervention—a multisensory scaffold of olfactory, proprioceptive, and vestibular stimulation during non-nutritive suck (NNS)-contingent caregiver’s recorded voice [14]. However, this intervention required skilled therapists to administer, and effects of the intervention on neural processing could have been due to several factors such as holding, scent cloth and vestibular stimulation.

The current study sought to isolate the effect of contingent exposure to a caregiver’s recorded voice, triggered by the infant’s own non-nutritive sucking (NNS), to determine whether this active, infant-controlled auditory stimulation enhances neural speech processing compared with passive, non-contingent exposure. To do so, a novel technology was used to deliver caregiver’s voice, that adjusted automatically to the preterm infants’ responses while they were still in their isolette or open crib. We hypothesized that contingent engagement with caregiver’s voice would increase speech-sound differentiation above passive exposure. Secondarily, we hypothesized this effect would not be impacted by ambient sound or the type of NICU environment during the intervention.

## 2. Materials and Methods

This single-blind randomized controlled trial was conducted from March 2024 to February 2026. Infants were enrolled from two Atlanta, Georgia NICUs: Emory University Hospital Midtown, a Level III referral center, and Grady Memorial Hospital, a Level III urban safety-net NICU. All analyses were conducted according to the intent-to-treat principle provided infants met the minimum protocol compliance. This protocol was reviewed and approved by Advarra (Approval No. Pro00070933) under a cede review agreement with the Emory University IRB (2023P005830). The study was registered on ClinicalTrials.gov (Identifier: NCT06063122) prior to beginning enrollment. Written informed consent was obtained from the parent(s) of each infant. A data safety monitoring board met regularly to oversee the study. In addition, the principal investigator, consistent with the conflict management plan, was blinded to group assignments, results, and analyses until an independent biostatistician reported results.

### 2.1. Eligibility

Infants were eligible for the study if they were born <35 weeks gestation, and between 32 0/7 weeks corrected gestational age (CGA) and 35 6/7 weeks CGA at study start and if their caregivers spoke English.

Exclusion criteria included mechanical ventilation via endotracheal tube, major congenital malformation, family history of genetic hearing impairment, use of sedative/seizure medication, and severe white matter injury. Infants who did not pass their newborn hearing screen were withdrawn from the study.

Eligible infants were randomized to the active intervention or passive control group using permuted-block randomization stratified by site with random block size generated by the statistical core. Dosage was two to three 20 min sessions daily, with a goal of delivering 20 sessions across 14–21 days. Compliance with the study protocol was set at a minimum of 12 sessions. Sessions were delivered by board-certified music therapists (MT-BC) or other trained study staff (NICU nurses, research coordinators) overseen by an MT-BC.

### 2.2. Study Protocol

Both groups received routine neonatal care practices without modifications for study procedures. Caregivers reported demographic information. Sessions were attempted directly before or after routine infant care. Before sessions, infant readiness and cardiorespiratory stability were assessed by study staff and bedside nurses. Sessions were discontinued if the infant did not spontaneously engage with the pacifier, fell asleep, or demonstrated autonomic instability (>2 apneas, bradycardia, or desaturation episodes). Consecutive sessions were >3 h apart.

### 2.3. Caregiver Voice Recording

Caregivers were recorded in a quiet room with protocolized coaching to elicit approximations of infant-directed speech and singing, as developed by Kjeldsen et al. (2025) [19]. Recordings were made using the smallTalk NICU Egg for Parents app (smallTalk, Chagrin Falls, OH, USA) and included lullabies, nursery rhymes, and children’s books.

### 2.4. Session Procedures

Infants in the control group received non-contingent, passive exposure to their recorded caregiver’s voice played via the smallTalk NICU egg (smallTalk, Chagrin Falls, OH, USA). This speaker is calibrated to limit play time to 20 min and produces sound intensities ≤ 55 dB(A). Infants in the intervention group received an active intervention where their caregiver’s voice recording was played contingently on their non-nutritive suck (NNS) using the smallTalk^®^ NICU Active eEgg^TM^ system and its algorithms (smallTalk, Chagrin Falls, OH, USA) which similarly limits sound intensities to <55 dB(A). The system consists of a reusable, cleanable Active Egg speaker device connected to a reusable, cleanable pacifier sensor, designed to fit into the back of a disposable pacifier (NICU Soothie, Philips Avent, Amsterdam, The Netherlands) and connected to the Egg speaker via Bluetooth. During the session, NNS provided an infant-controlled mechanism for administration of the caregiver’s voice: when the infant’s NNS met an individually calibrated pressure threshold, the caregiver’s voice recording played for 10 s before stopping, requiring the infant to again meet their threshold to reactivate the recording. NNS-contingent reinforcement paradigms have been used to demonstrate reliable infant engagement and learning in response to salient stimuli [20,21,22,23].

During the session, the pacifier sensor continuously measured and transmitted the infant’s NNS strength to the Active Egg via Bluetooth. Session data were collected using the smallTalk Therapy App (smallTalk, Chagrin Falls, OH, USA), also connected to the Active Egg via Bluetooth. Because playback in the intervention condition was contingent on threshold-level NNS and capped at 10 s increments requiring reactivation, cumulative voice exposure time within an active session was limited relative to the control condition.

### 2.5. Masking Procedures

Family caregivers were masked to group assignment with staff caregivers masked to the extent feasible within the clinical environment. The setup of all sessions, regardless of the participant’s assigned group, included the pacifier-mounted sensory such that the physical setup was visually indistinguishable between groups. The primary difference between conditions—that infants in the intervention were required to suck consistently to activate their caregiver’s voice recording—was not disclosed to staff caregivers. Research staff positioned themselves to minimize incidental observation of infant sucking behavior during sessions; however, complete visual obstruction was not implemented as restricting nursing access to bedside would have been clinically inappropriate and potentially unsafe. Given the routine demands of the NICU environment for staff, incidental unmasking through observation of sucking behavior was considered unlikely to influence care or introduce bias given the outcomes being EEG-derived.

### 2.6. Environmental Sound Levels

Sound levels were measured throughout each session via an Extech 407760 Sound Level Datalogger (sampling rate 1s, dB(A) weighting) (Extech Instruments, Nashua, NH, USA).

Data collected included gestational age at birth, postmenstrual age at first assessment, parent-identified race and ethnicity, maternal education, bed type, and respiratory support status.

### 2.7. Outcome Measures

**Figure 1:**
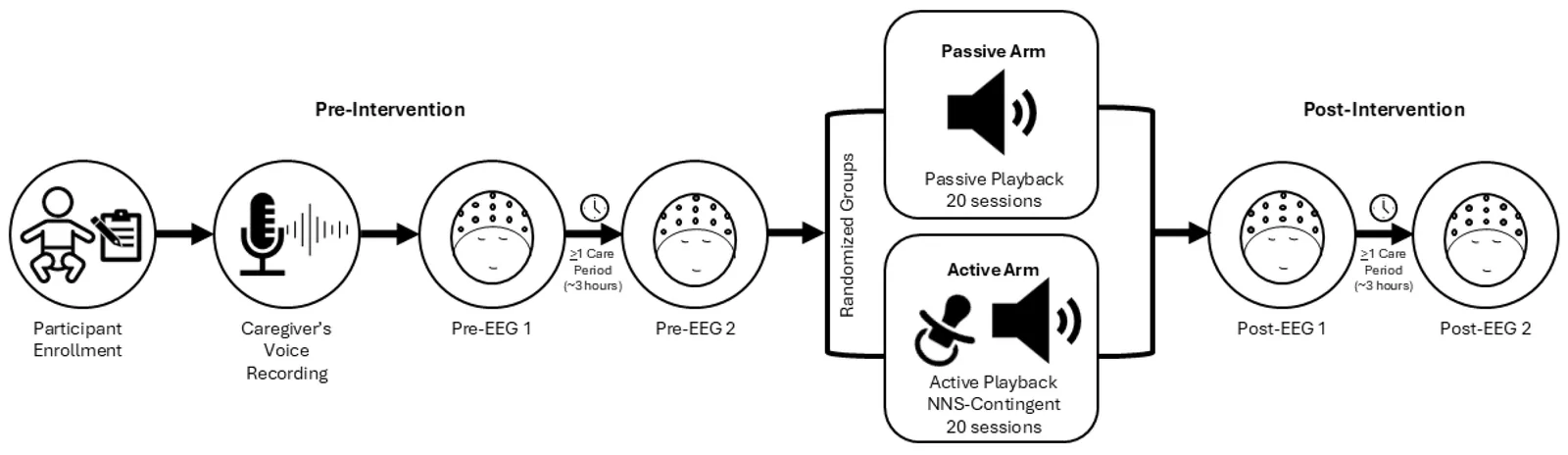
Participation flowchart.

To increase the robustness of EEG findings, two separate data collections were performed at baseline and post intervention, at a minimum of one care period (~3 h) apart, with averaging of amplitudes after extraction. A flowchart depicting an individual participant’s study involvement is displayed in Figure 1. Auditory event-related potentials (ERP) were collected using time-locked EEG using a 64-channel Ag/AgCl electrode net (eego^TM^ v.1.10.2 with eego^TM^64 amplifier, EE-225, and **wave**guard^TM^ neonatal nets, ANT Neuro, Berlin, Germany) at 1000 Hz with filters set to 0.1–400 via eego software. Electrodes were referenced to Cz during recording and later re-referenced offline to an average reference. Infants’ head circumference was ≥25 cm to ensure adequate scalp contact. Electrode impedance was kept to <40 kΩ.

Even though only one contrast was of interest as prespecified (see below) to minimize comparisons, six computer-generated female American English speech sounds (/ba/, /bu/, /da/, /du/, /ga/, /gu/) were delivered to minimize habituation. They were presented binaurally in free field at 60 dB SPL (±5 dB) via a neodymium speaker placed 10 cm from the infant’s head, 25 times each in random order with randomized inter-onset interval (1.3–2.3 s) [14]. Custom Raspberry Pi (Raspberry Pi, Cambridge, UK) software delivered stimuli and provided time-locked EEG markup. The ~8 min paradigm was conducted while infants were awake or drowsy.

Data were filtered using a 0.3–40 Hz bandpass filter and 60 Hz notch filter to remove line artifacts. Independent component analysis (ICA) was used for automatic artifact detection and removal (i.e., eye movement, blinking, mouth movement, etc.) followed by reconstruction with ICA-label using package *mne* (v.1.9.0). Epochs were segmented from −200 to 1000 ms from stimulus onset. Data for electrodes with poor signal were interpolated; segments with >12 bad electrodes were rejected.

### 2.8. Statistical Analysis

Sample size was determined a priori assuming α = 0.05, 80% power, with balanced group allocation, and a conservative R^2^ of 0.5 based on preliminary pilot data; to detect a minimum effect size of d = 0.29, the required sample size was N = 190. As pre-specified in the analysis plan based on pilot data, we conducted analyses focusing on the /d/_/g/ consonant contrast using an ANCOVA framework. Separate linear models were fit for each scalp location (T5 and T6), with post-intervention EEG amplitude as the outcome and baseline EEG amplitude included as a covariate to adjust for pre-intervention differences. Treatment group (intervention vs. control) was included as the primary predictor of interest. These models estimated the adjusted mean difference in post-intervention EEG between groups at each scalp location. Model assumptions were evaluated using standard residual diagnostics, which did not indicate major violations. In sensitivity analyses, additional covariates (gestational age at birth and maternal education) were included to assess robustness of the estimated treatment effect. Additional models incorporating interaction terms were used to evaluate whether the treatment effect varied as a function of environmental factors (sound level and setting) and clinical variables (respiratory status and neuroimaging severity). Estimates are reported with 95% confidence intervals and corresponding *p*-values. All analyses were completed using R (v.4.6.0). 

## 3. Results

A total of 214 infants were enrolled in the study, with 191 completing all study procedures (see Supplemental Figure S1, CONSORT diagram). Participant characteristics are summarized in Table 1, showing demographic and clinical variables to be well balanced between groups. The average number of usable EEG trials did not differ between intervention and control groups at either pre- or post-intervention assessments. Protocol adherence was high, with 97.1% fidelity to session delivery.

### 3.1. Primary Intervention Effect

After controlling for pre-intervention EEG, analysis revealed a statistically significant difference in post-intervention auditory ERP responses at the left temporal scalp location (T5) between the intervention and control groups (β = 0.023, 95% CI [0.002, 0.044], *p* = 0.036) and a borderline significant effect at the right temporal scalp location (T6) (β = 0.022, 95% CI [0.000, 0.045], *p = 0*.054). This corresponds to a small effect at T5 (*d* = 0.31) and at T6 (*d* = 0.28).

Sensitivity analyses including gestational age at birth and maternal education as covariates yielded similar findings at T5 (β = 0.024, 95% CI [0.002, 0.045], *p* = 0.032) and T6 (β = 0.024, 95% CI [0.001, 0.047], *p* = 0.038).

### 3.2. Effect of Bed and Room Type

No statistically significant interactions were found between treatment group and bed type (isolette versus open crib) or room type (open bay, semi-private, and private room) (all *p* > 0.05; Table 2). Mean and maximum sound levels across the session were not different between groups, bed type, or room type and did not demonstrate an interaction with post-intervention speech-sound differentiation ability.

### 3.3. Exploratory Secondary Analyses of Clinical Variables

Follow-up analyses considered whether additional clinical variables and outcomes affected models and examined (1) whether the estimated intervention effect changed and (2) whether the clinical variables were associated with the outcome. These analyses found no significant main effect of respiratory status (*p* = 0.471) or neuroimaging severity (*p* = 0.649), and the intervention effect persists even when controlling for these two variables (Table 3).

Overall, these findings provide evidence of a significant enhancement in neural speech processing, as measured by auditory ERP, following contingent caregiver voice exposure compared to passive exposure in preterm infants.

## 4. Discussion

This randomized controlled trial demonstrates that alone, contingent, NNS-activated exposure to a caregiver’s recorded voice enhances neural speech sound differentiation in hospitalized preterm infants compared to those exposed passively. Infants in the intervention group showed significantly greater post-intervention auditory ERP responses at the left temporal scalp location, with a comparable effect at the right temporal scalp location when controlling for gestational age and maternal education level. These findings correspond to a small but meaningful effect at both locations. Notably, the observed effect sizes at T5 and T6 (*d* = 0.31 and 0.28, respectively) closely approximated the minimum detectable effect size of *d* = 0.29 specified in the a priori power analysis. Thus, the observed effects were consistent with the magnitude the study was designed to detect. The comparatively stronger effect observed at the left temporal scalp location is consistent with established left-hemispheric lateralization for speech and language processing [24,25,26], potentially reflecting a preferential engagement of the neural networks biased toward linguistic processing following active engagement with caregiver’s voice recording at this early stage of neurodevelopment. Importantly, these findings extend prior work on the MIND intervention [14], demonstrating that active engagement with the recorded caregiver’s voice through contingent NNS is an independent driver of neural change.

### 4.1. Contingency

The contingent reinforcement approach utilized in this study drew on a well-established literature demonstrating infants’ capacity for reliable engagement with and learning from salient auditory stimuli [14,16,17,18]. The present study extends this paradigm and establishes a mechanism or early marker of language development; however, the long-term language outcomes were not probed by the current design. As such, these findings establish a plausible neural mechanism by which contingent auditory engagement may support downstream language development, but they do not themselves resolve whether the observed enhancement in neural processing translates into measurable improvements in language long-term. Future follow-up studies are needed to close that loop.

Notably, the contingency requirement in this study structurally limited cumulative voice exposure time within an active session relative to control sessions, in which the recording played continuously; infants in the intervention group therefore received less cumulative exposure to their caregiver’s voice recording than controls. Despite this reduced quantity of auditory input, the intervention group demonstrated significantly greater enhancement in cortical speech sound differentiation. This pattern suggests that the way the caregiver’s voice was delivered—specifically, its contingency on the infant’s own active behavior—may matter more to neural processing than the total duration of exposure itself. Future studies incorporating objective measurement of cumulative exposure duration in each condition would help further quantify the relationship between dose, contingency, and speech-processing outcomes.

### 4.2. Infant-Directed Voice

The caregiver voice recordings that were produced for this study followed a coached protocol designed and validated to elicit the acoustic characteristics of infant-directed speech and infant-directed singing, rather than neutral or conversational speech [19]. Infant-directed voice is acoustically and prosodically different from adult-directed voice (exaggerated pitch contours, slower tempo, greater rhythmic regularity) [27], and previous research has shown that preterm infants differentially process infant-directed and adult-directed recordings [19]. The characterizable features of infant-directed voice have previously been associated with heightened infant attention and early learning of speech sounds in term infants [27,28]. It is possible that the improvement in speech sound differentiation observed in the present study may be a reflection of exposure to not only the caregiver’s voice, but the actual voice characteristics that are maximally attended to by the immature brain and auditory system. Future work directly comparing infant-directed recordings to adult-directed recordings, as well as those recorded in the presence and absence of an infant, within a contingent paradigm, would help differentiate between the relative contributions of voice familiarity, prosody, and contingency to these outcomes.

### 4.3. Gestational Age and Maternal Education

Sensitivity analyses including gestational age at birth and maternal education level strengthened, rather than weakened, the observed treatment effect. Gestational age is a well-established determinant of auditory system maturity: infants at increasingly lower GA show decreased amplitude and increased latency in auditory brainstem response [29,30]. Its emergence as a significant covariate is therefore consistent with the broader literature on preterm auditory development, reflecting underlying biological variability rather than a limitation of the contingent-voice intervention. The significance of maternal education also aligns with a substantial body of work linking socioeconomic and educational factors to early language development and language outcomes [31]. Speech quantity, quality, and complexity as well as the home language environment have all been implicated as pathways that could possibly influence an infant’s responsiveness to speech stimuli [27,32]. Its association with the post-intervention EEG suggests that even a controlled, standardized intervention does not fully counter the influence of the broader environmental and socioeconomic factors associated with maternal education. This underscores the importance of considering family context in any NICU-initiated language interventions.

### 4.4. Signal Audibility and Acoustic Safety

In addition to the primary outcomes of interest, another important question motivating this study was whether infants across differing levels of respiratory support could reliably perceive their caregiver’s voice recording against the background noise level of their own ambient equipment noise. Because respiratory support modalities such as CPAP and high-flow nasal cannula generate substantial ambient noise at bedside [33], a potential concern was the possibility that the equipment noise would mask or otherwise limit an infant’s exposure to the recording, independent of whether the recording was delivered at a safe volume. Analyses showed that respiratory support status did not significantly interact with the treatment effect, suggesting that infants were able to sufficiently perceive and benefit from exposure to their caregiver’s voice recording regardless of the ambient noise associated with their respiratory equipment. Sound levels were closely monitored throughout all sessions and mean session noise levels remained below the limit considered safe for the developing auditory system at the hourly L_10_ level (≤55 dBA) [34]. Though maximum noise levels recorded during sessions exceeded current recommendations for the 1 s L_max_ (<70 dB(A)) [34], maximum noise exposures were not associated with the neural processing of speech sounds. Importantly, these noises were produced by sources external to the session equipment.

The absence of significant interactions by bed or room type also suggests that the intervention was tolerated consistently across the variable acoustic and physical environments often found in the NICU. When taken together, these findings support the feasibility and safety of infant-activated, contingency-based auditory interventions in preterm infants as young as 32 weeks corrected gestational age.

## 5. Limitations

Because the control group received full passive exposure to their caregiver’s voice recording rather than no auditory stimulation, the estimated effect size in this study likely represents a conservative comparison between two active forms of exposure rather than the fully attainable effect of contingent exposure relative to auditory deprivation. Despite this conservative framing, the observed effect remained statistically significant and meaningful. While complete masking of bedside staff was not achievable in the clinical NICU setting, the standardized session appearance and the practical demands on nursing staff minimized the likelihood of differential awareness of group assignment. Finally, because nearly all of the infants received the full number of sessions, the available data provide limited statistical information to estimate a dose–response relationship. Future work should evaluate different doses to determine whether a dose–response relationship exists.

## 6. Conclusions

This randomized controlled trial provides evidence that contingent, infant-activated exposure to their caregiver’s voice recording enhances neural speech sound differentiation in hospitalized preterm infants. The intervention was both feasible and acoustically safe across a range of clinical and environmental conditions common in the NICU, including various levels of respiratory support, bed type, and room configuration. This supports its potential for broader implementation, particularly in NICUs where caregivers face barriers to physical presence at the bedside. While the present findings establish a proximal neural mechanism linking active auditory engagement with enhanced neural processing of speech sounds, longitudinal follow-up is needed to determine whether these early neural changes translate to measurable improvements in language long-term.

## Figures and Tables

**Table 1:** Participant demographic characteristics.

Characteristic	Control (n = 94)	Intervention (n = 97)	Full Cohort (n = 191)
**Sex**			
Female	50 (53.2%)	51 (52.6%)	101 (52.9%)
Male	44 (46.8%)	46 (47.4%)	90 (47.1%)
**GestationalAge (Weeks)**			
Mean (SD)	31.3 (2.19)	31.5 (2.40)	31.4 (2.29)
Median [Min, Max]	31.7 [25.7, 34.6]	31.9 [23.3, 34.4]	31.9 [23.3, 34.6]
**Race**			
American Indian or Alaska Native	1 (1.1%)	0 (0%)	1 (0.5%)
Asian	2 (2.1%)	1 (1.0%)	3 (1.6%)
Black or African American	74 (78.7%)	86 (88.7%)	160 (83.8%)
More than One Race	1 (1.1%)	3 (3.1%)	4 (2.1%)
Native Hawaiian or other Pacific Islander	1 (1.1%)	1 (1.0%)	2 (1.0%)
Other	3 (3.2%)	1 (1.0%)	4 (2.1%)
Unknown	1 (1.1%)	0 (0%)	1 (0.5%)
White	11 (11.7%)	5 (5.2%)	16 (8.4%)
**Maternal Education (Years)**			
Mean (SD)	14.0 (2.85)	14.0 (2.85)	14.0 (2.85)
Median [Min, Max]	12.0 [11.0, 20.0]	12.0 [9.00, 20.0]	12.0 [9.00, 20.0]
Missing	5 (5.3%)	4 (4.1%)	9 (4.7%)
**Corrected Gestational Age at Study Initiation**			
Mean (SD)	33.6 (0.88)	33.9 (0.83)	33.8 (0.86)
Median [Min, Max]	33.7 [32.1, 35.6]	33.9 [32, 35.9]	33.9 [32, 35.9]
**Respiratory Status at Study Initiation**			
Room Air	58 (61.7%)	55 (56.7%)	113 (59.2%)
Nasal Cannula	1 (1.1%)	2 (2.1%)	3 (1.6%)
High-Flow Nasal Cannula	18 (19.1%)	25 (25.8%)	43 (22.5%)
CPAP	17 (18.1%)	15 (15.4%)	32 (16.8%)
**Neuroimaging Findings**			
Within Normal Limits	79 (84.0%)	82 (84.5%)	161 (84.3%)
Abnormal Findings (Grade I-II IVH)	15 (16.0%)	15 (15.5%)	30 (15.7%)
**Discharge Weight (Grams)**			
Mean (SD)	2720 (611)	2640 (600)	2680 (605)
Median [Min, Max]	2610 [1710, 4570]	2570 [1840, 5010]	2600 [1705, 5010]
**Number of Sessions Completed**			
Mean (SD)	19.22 (1.69)	18.80 (2.19)	19.01 (1.97)
Median [Min, Max]	20 [12, 20]	20 [12, 20]	20 [12, 20]
**Bed Type**			
Open Crib	55 (58.5%)	69 (71.1%)	124 (64.9%)
Isolette	39 (41.5%)	28 (28.9%)	67 (35.1%)
**Room Type**			
Private Room	2 (2.1%)	3 (3.1%)	5 (2.6%)
Semi-Private Space	27 (28.7%)	28 (28.9%)	55 (28.8%)
Open Bay	65 (69.2%)	66 (68.0%)	131 (68.6%)
**Mean Session Sound Level,** (dB(A))			
Median [Min, Max]	49.0 [44.9, 53.42]	49.0 [43.4, 55.56]	49.0 [43.4, 55.56]
**Maximum Sound Level,** (dB(A))			
Median [Min, Max]	74.2 [69.5, 101.9]	73.4 [66.7, 83.0]	74.2 [66.7, 101.9]

Note: Severe white matter injury was an exclusion for the study; IVH: intraventricular hemorrhage.

**Table 2:** Effect of bed and room type.

Variable	Scalp Location	Adjusted Intervention Main Effect, β (95% CI)	*p*-Value	Interaction Association with Outcome β (95% CI)	*p*-Value
**Bed Type**	T5	0.031 (−0.005, 0.067)	0.093	−0.015 (−0.06, 0.03)	0.513
Room Type *		0.031 (0.005, 0.056)	0.018	-	-
Private/Open Bay		-	-	0.013 (−0.123, 0.148)	0.853
Semi-Private/Open Bay		-	-	−0.030 (−0.077, 0.017)	0.207
**Average Sound Level**		0.023 (0.002, 0.044)	0.036	0.005 (−0.006, 0.017)	0.341
**Maximum Sound Level**		0.024 (0.002, 0.045)	0.031	−0.001 (−0.008, 0.005)	0.686
**Bed Type**	T6	0.050 (0.012, 0.088)	0.011	−0.044 (−0.091, 0.004)	0.071
Room Type *		0.027 (−0.001, 0.054)	0.055	-	-
Private/Open Bay		-	-	0.001 (−0.143, 0.146)	0.986
Semi-Private/Open Bay		-	-	−0.017 (−0.067, 0.033)	0.501
**Average Sound Level**		0.022 (0, 0.045)	0.052	0.009 (−0.003, 0.021)	0.142
**Maximum Sound Level**		0.023 (0, 0.046)	0.053	0.001 (−0.008, 0.005)	0.686

* Note: Room type was modeled as a three-level categorical variable with “Open Bay” as the reference category. The Adjusted Intervention Effect reflects the intervention effect among infants in Open Bay rooms while the Interaction column reflects the difference in the intervention effect for each comparison group (Private, Semi-Private) relative to Open Bay.

**Table 3:** Exploratory secondary analyses of clinical variables at T5 scalp location.

Covariate Added to Model	Adjusted Intervention Effect, β (95% CI)	*p*-Value	Covariate Association with Outcome *	*p*-Value
Respiratory Status	0.025 (0.003, 0.046)	0.023	Partial F (3, 184) = 0.84	0.471
Neuroimaging Severity	0.023 (0.001, 0.044)	0.036	β = −0.007 (−0.036, 0.022)	0.649

* Note: Each row reflects a separate ANCOVA model adding the listed clinical variable as a covariate to the model specification from the primary analysis (Post-EEG ~ Group + Pre-EEG + Covariate). Respiratory status association with the outcome is summarized by partial F-test across all levels; neuroimaging severity, a binary covariate, is summarized by its single-term estimate.

## Data Availability

Data may be provided upon reasonable request.

## Supplementary Materials

The following supporting information can be downloaded at: https://www.mdpi.com/article/10.3390/jcm15176880/s1, Figure S1: CONSORT Flow Diagram.
