# Supplementary material for: Recorded Contingent Caregiver Voice Independently Improves Neural Speech Processing in Hospitalized Preterm Infants: A Multisite Randomized Controlled Trial
Source: J Clin Med. 2026 Sep 5;15(17):6880. doi: 10.3390/jcm15176880 (PMC13565975; doi:10.3390/jcm15176880)
Supplement: Supplementary file 1 [file jcm-15-06880-s001.zip › jcm-4505836-supplementary.pdf]

Supplemental Figure S1. CONSORT Flow Diagram

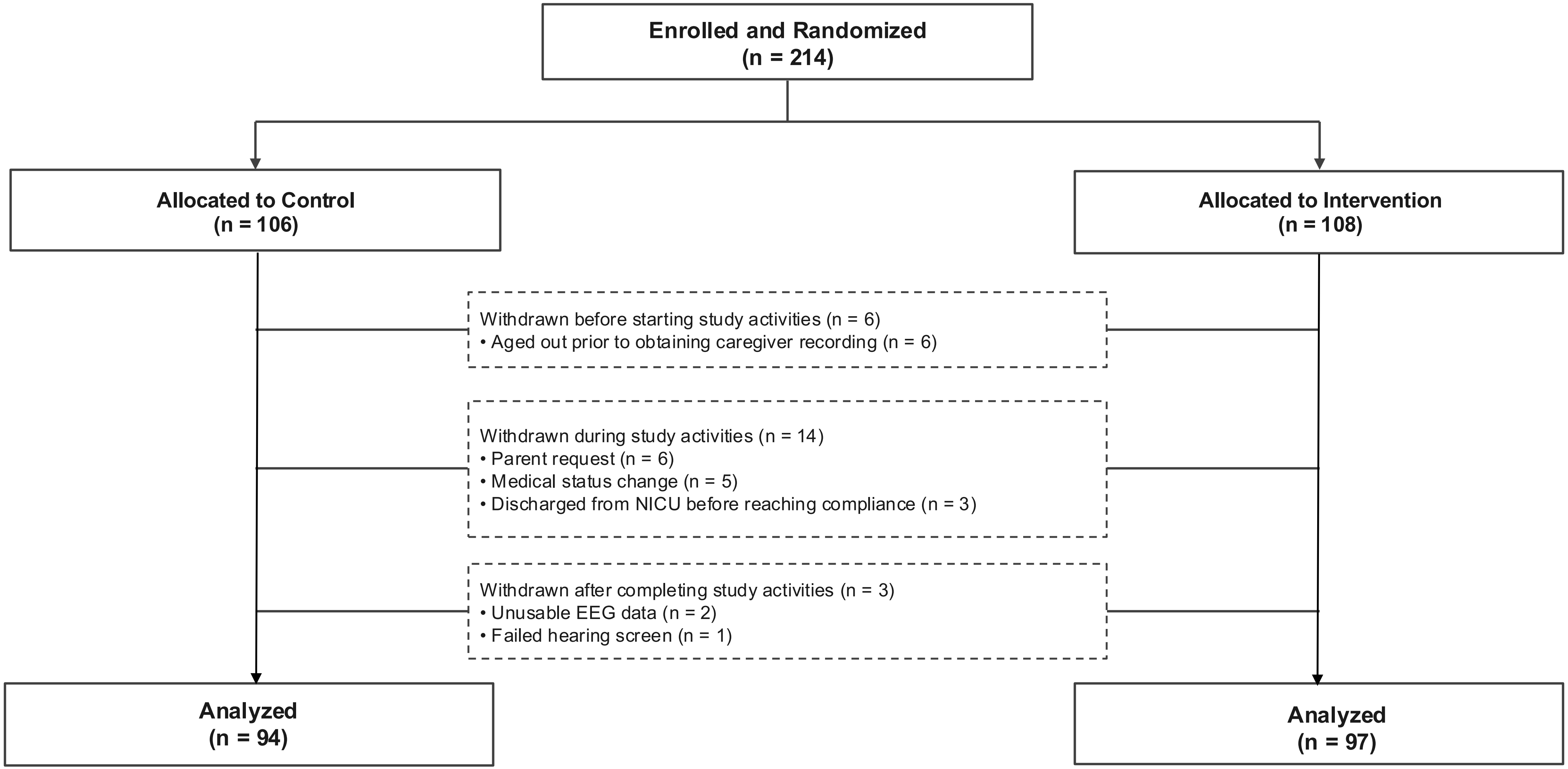

Note: Withdrawal reasons were recorded in aggregate across intervention and control arms and are displayed once, applying toboth columns. Final analyzed n reflects group-specific totals.
